# Lateral epicondyle to the joint line distance is a precise landmark for determination of an accurate knee joint line: an observational retrospective study

**DOI:** 10.1186/s40634-023-00621-z

**Published:** 2023-06-08

**Authors:** Wazzan S. Aljuhani, Abdullah A. Alsaeed, Maali O. Alrashed, Abdullah M. Alanazi, Mohammed J. Alsalman

**Affiliations:** 1 Department of Orthopedic Surgery, Ministry of the National Guard – Health Affairs, Riyadh, Saudi Arabia; 2 King Abdullah International Medical Research Center, Riyadh, Saudi Arabia; 3 King Saud Bin Abdulaziz University for Health Sciences, Riyadh, Saudi Arabia; 4 Department of Surgery, Unaizah College of Medicine and Medical Sciences, Qassim University, Buraydah, Saudi Arabia; 5 Department of Radiology, Ministry of the National Guard – Health Affairs, Riyadh, Saudi Arabia

**Keywords:** Knee replacement surgery, Persistent pain, Recognizable anatomical landmarks, Accurate knee joint line estimation, Varus malalignment

## Abstract

**Purpose:**

To assess a quantitative and reproducible association between the position of the knee joint line and recognizable anatomical landmarks around it in order to help in restoring joint line in arthroplasty cases.

**Methods:**

Magnetic resonance imaging (MRI) of 130 normal knees were investigated. Anatomical measurements of the knee joint distances on the obtained planes were performed manually by distance measurements using a ruler tool, followed by 6 anatomical bony landmarks determination about the knee to identify the joint line which included the joint line, medial epicondyle, lateral epicondyle, medial flare, lateral flare, and proximal tibiofibular joint. The entire process was examined twice by two independent fellowship trained musculoskeletal radiologists, with a 2-week interval between the first and second sets of readings.

**Results:**

The lateral epicondyle to the joint line of the knee (LEJL) could be a reliable landmark for accurate distance measurements for the knee joint line level, with an absolute distance of 24.4 ± 2.8 mm. The analysis showed that the femorotibial ratio between the LEJL and proximal tibiofibular joint (PTFJ) was 1.0 (LEJL/PTFJJL = 1.0 ± 0.1), confirming the location of the knee joint at the midpoint between the lateral epicondyle and PTFJ, revealing two identifiable landmarks.

**Conclusions:**

LEJL is the most precise landmark for determination of an accurate knee joint line because the knee is located at the midline between the lateral epicondyle and PTFJ. These reproducible quantitative relationships can be widely employed in various imaging modalities to help restore the knee JL in arthroplasty surgeries.

## Background

Determination of the knee joint line (JL) position is essential in total knee replacement surgery, specifically in revision procedures. Unsuccessful restoration of knee JL could cause worse clinical and biomechanical outcomes. For example, raising the JL beyond 8 mm would result in mid-flexion instability and worse clinical outcomes [7, 8, 10, 13]. These challenges call for a need for reliable landmarks for precise knee JL distance assessment. However, there is no standard technique for measuring the JL position. The most widely used anatomical points are the femoral epicondyles, adductor tubercle, fibular head, and patella; the JL can be appropriately determined by measuring the distances between these anatomical points [6, 15].

Surgeons use different measurement approaches; some measure the distance between the adductor tubercle and the JL, whereas others rely on the lateral epicondyle of the distal femur to determine the JL [5]. Meanwhile, in the anteroposterior (AP) view, surgeons prefer the epicondyles as references. Alternatively, utilization of the contralateral knee, if not replaced, could be used to measure the JL of the index knee [14]. There are no specific criteria for a perfect radiological view [5]. Hence, restoration of the knee JL during revision surgery is difficult [2].

Similarly, previous studies have been conducted to establish an association between the fibular styloid and tibial plateau; however, research and observations on the fibular head are still lacking in terms of consensus [13, 14]. Moreover, during the proximal tibial cut, the fibular head maybe removed intraoperatively. Therefore, the head of the fibula is considered extremely inconstant and independent as an anatomical landmark [1, 4, 15].

In a study in the USA in 2016, the magnetic resonance imaging (MRI) data of 50 normal human adult knees were randomly selected for JL estimations. The results indicated that the JL was at an equal distance between the lateral femoral epicondyle and the proximal tibiofibular joint (PTFJ), which made it a reproducible reference for JL restoration [11]. However, only 50 images were utilized in a specific population which cannot be generalized to the whole population [11].

Therefore, the aim of the current study was to assess with MRI evaluation a quantitative and reproducible association between the position of the knee joint line and recognizable anatomical landmarks around it as distances from bony landmarks to joint line seemed to be affected by gender and ethnicity [11].

## Methods

### Ethical approval

Ethical approval was obtained from King Abdullah International Medical Research Center, Riyadh, Saudi Arabia with the study number (RC17 / 273 / R). Anonymity and confidentiality of participants were ensured throughout the study. Due to the nature of the study, informed consent was waived.

### Study participants

This observational retrospective cohort study randomly selected adult patients who underwent knee MRIs from June 1, 2016, to July 31, 2017, performed at our institution, with normal knee results. A total of 130 patients were included for the measurements of anatomical landmarks of the knee JL.

### Exclusion/inclusion criteria

Knee joints with arthritis, ligament and meniscal injuries, and osteochondral defects were excluded from the study. After two fellowship-trained musculoskeletal radiologists ensured that the MRI fulfilled the criteria for each anatomical landmark measured using a distance ruler tool included as part of the MRI software, the participants’ data were enrolled in the study (Table 1).

**Table 1:** Inclusion and exclusion criteria

Inclusion Criteria	Exclusion Criteria
Normal knees with identifiable landmarks	Knee arthritis, ligmanets injuries, meniscus injuries, chondral defects, presence of hardware or fracture

### Knee anatomical landmarks measurements

The following anatomical landmarks were identified on MRI, according to the study reported by Pereira et al. [11]:

#### Landmarks in the coronal section (Fig. 1a)

**Fig. 1:**
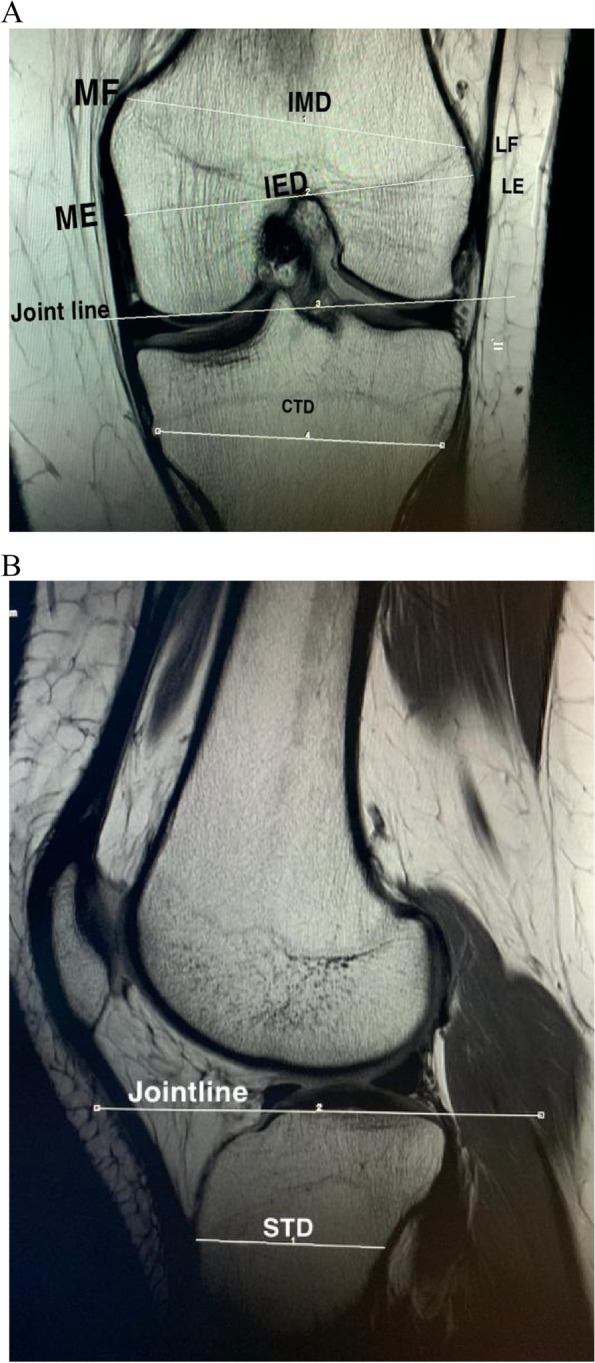
**a** Landmarks in the coronal section. **b** Landmarks in the sagittal section

1. Knee JL was determined through a line that connects the most distal points of the medial and lateral femoral condyles.
2. The medial epicondyle (ME) was defined as the point where the medial collateral ligament originated.
3. The lateral epicondyle (LE) was defined as a bony projection on the lateral side of the distal femur where the lateral collateral ligament originated.
4. The point where the medial condylar cortex and the medial metaphyseal flare joins was defined as the medial flare (MF).
5. Lateral flare (LF) was defined as the joining point between the lateral femoral metaphyseal flare and lateral condylar cortex.
6. The middle horizontal part of the proximal tibiofibular joint was considered the PTFJ.

#### Landmarks in the sagittal section (Fig. 1b)

1. The most proximal angle of the connection between the tuberosity and anterior cortex of the tibia and the most proximal point of the patellar tendon insertion was used if this angle could not be identified and was considered the tibial tubercle (TT).

The exact steep distance between JL and every anatomical point was measured and documented.

### Measurements using the coronal section

A. The distance between the ME and LE is known as the surgical transepicondylar axis, which reflects the interepicondylar diameter of the femur (IED).
B. The distance between the MF and LF indicates the intermetaphyseal diameter of the femur (IMD).
C. The tibial width at the level of the PTFJ and vertical to the tibial shaft reflects the coronal tibial diameter (CTD).

### Measurements using the sagittal plane

A. The tibial diameter at the level of the TT and vertical to the tibial shaft reflects the sagittal tibial diameter (STD).

The exact distances to the respective bony diameters were normalized by dividing the suitable diameter by the corresponding accurate distance (e.g., CTD: PTFJJL) to control the variations between different sexes [1, 15]. These were termed "femoral ratios" or "tibial ratios " We calculated the overall spatial relationship of the femoral and tibial landmarks in relation to the knee JL by determining the ratios between the absolute femoral and tibial distances. These were termed "femorotibial ratios."

Two independent observers measured the landmarks of each participant. A total of 40 random knee MR images were used to assess intra- and inter-observer reliabilities before proceeding. The measurements were repeated twice, 2 weeks apart.

The measurements were performed manually on the MRIs with a measure distance ruler tool, specifically designed for such estimations and included as part of the MRI software.

Patients’ anonymity was ensured throughout the study by using serial numbers instead of their names or IDs.

The requirement for informed consent was waived owing to the retrospective nature of the study.

### Statistical analyses

Statistical analysis was performed using Statistical Analysis System (SAS, version 9.4). Numerical variables were represented as mean and standard deviation (SD). To determine the inter- and intra-observer reliability, intraclass correlation coefficients were calculated with a 95% confidence interval. The mean measurements were reported, and the error was reported as SD. Statistical significance was determined via a two-tailed student's t-test, and statistical significance was set at *p* < 0.05.

## Results

Both observers found a very strong positive correlation between the measurements on days 1 and 2 in all factors except the MEJL. There was excellent inter-observer reliability in the MEJL, LEJL, MFJL, LFJL, PTFJJL, TTJL, STD, IED, IMD, and CTD (Tables 2 and 3).

**Table 2:** Intra-observer reliability

	**Measurements at day 1 (*n* = 40)** **Mean ± SD**	**Measurements at day 14 (*n* = 40)** **Mean ± SD**	**Intraclass Correlation**
MEJL	20.80 ± 2.66	19.95 ± 2.57	0.312
LEJL	23.67 ± 2.31	24.58 ± 2.52	0.681
MFJL	38.06 ± 3.47	38.27 ± 3.55	0.918
LFJL	30.61 ± 2.87	30.66 ± 3.26	0.779
PTFJJL	26.70 ± 2.43	25.96 ± 3.60	0.784
TTJL	30.83 ± 4.85	29.25 ± 4.25	0.880
IED	76.59 ± 5.78	76.64 ± 5.69	0.996
IMD	72.23 ± 5.74	72.11 ± 5.62	0.987
CTD	59.46 ± 6.32	62.56 ± 5.17	0.788
STD	38.24 ± 4.61	38.73 ± 5.15	0.876

*JL* Joint line, *MEJL* Medial epicondyle to the knee JL, *LEJL* Lateral epicondyle to the knee JL, *LFJL* Lateral flare to the knee JL, *MFJL* Medial flare to the knee JL, *PTFJJL* Proximal tibiofibular joint to the knee JL, *TTJL* Tibial tubercle to the knee JL, *IED* Interepicondylar diameter, *IMD* Intermetaphyseal diameter, *CTD* Coronal tibial diameter, *STD* Sagittal tibial diameter, *SD* Standard deviation

**Table 3:** Inter-observer reliability

	**First reader** **(*n* = 40)** **Mean ± SD**	**Second reader** **(*n* = 40)** **Mean ± SD**	**Intraclass Correlation**
MEJL	20.37 ± 2.019	20.50 ± 1.84	0.946
LEJL	24.13 ± 2.12	23.63 ± 2.12	0.968
MFJL	38.17 ± 3.38	38.38 ± 2.80	0.981
LFJL	30.63 ± 2.77	31.03 ± 2.66	0.960
PTFJL	26.33 ± 2.80	26.78 ± 2.07	0.947
TTJL	30.04 ± 4.36	30.62 ± 3.92	0.960
IED	76.61 ± 5.72	77.62 ± 4.41	0.949
IMD	72.17 ± 5.64	71.83 ± 4.10	0.937
CTD	61.01 ± 5.38	60.98 ± 4.28	0.931
STD	38.49 ± 4.61	38.26 ± 38.26	0.981

*JL* Joint line, *MEJL* Medial epicondyle to the knee JL, *LEJL* Lateral epicondyle to the knee JL, *LFJL* Lateral flare to the knee JL, *MFJL* Medial flare to the knee JL, *PTFJJL* Proximal tibiofibular joint to the knee JL, *TTJL* Tibial tubercle to the knee JL, *IED* Interepicondylar diameter, *IMD* Intermetaphyseal diameter, *CTD* Coronal tibial diameter, *STD* Sagittal tibial diameter, *SD* Standard deviation

The final analysis involved the MRI of normal individuals (*n* = 130); males, *n* = 79 (60.8%); females, *n* = 51 (39.2%); mean age ± SD: 29.33 ± 8.5 years. Seventy (53.8%) MRI images were from the right knee, and 60 (46.2%) were from the left knee.

The absolute distances of the measured anatomical landmarks to the knee JL and femoral and tibial diameters are shown in Tables 3 and 4. No statistically significant differences between the femoral and tibial diameters were observed between the sexes (*p* > 0.05). The femorotibial ratio relationships between the knee JL and femoral and tibial landmarks are shown in Tables 4 and 5.

**Table 4:** Absolute distance from each anatomical landmark to the knee JL in the study participants (*N* = 130)

Current study				Pereira et al. [9]
Mean ± SD, mm				Mean ± SD, mm
Distance	Males	Females	Overall	
MEJL	19.2 ± 2.3	17.7 ± 2.5^a^	18.6 ± 2.5	27.6 ± 3.2
LEJL	25.6 ± 2.4	22.5 ± 2.3^a^	24.4 ± 2.8	23.6 ± 2.3
MFJL	39.7 ± 2.9	34.5 ± 4.1^a^	37.7 ± 4.3	41.5 ± 4.0
LFJL	31.5 ± 2.6	28.1 ± 3.7^a^	30.2 ± 3.5	35.1 ± 3.8
PTFJJL	26.6 ± 2.9	24.5 ± 2.9^a^	25.8 ± 3.1	22.2 ± 3.2
TTJL	30.3 ± 4.4	27.4 ± 7.5	29.1 ± 6.0	20.9 ± 4.4

^a^ Statistically significant difference at *p* < 0.05

**Table 5:** Absolute femoral and tibial diameters in the study participants (*N* = 130)

Mean ± SD, mm			
Diameter	Males	Females	Overall
IED	80.6 ± 4.0	71.1 ± 3.9^a^	76.9 ± 6.1
IMD	76.1 ± 4.0	67.4 ± 3.8^a^	72.7 ± 5.7
CTD	66.2 ± 6.2	58.7 ± 3.8^a^	63.3 ± 6.5
STD	41.2 ± 4.9	37.1 ± 4.7^a^	39.6 ± 5.2

^a^Statistically significant difference at *p* < 0.05

Table 4 shows the absolute distance between the knee JL and each anatomical landmark. Except for TTJL, all measured distances were significantly different between the male and female participants (*p* < 0.05).

In addition, the absolute femoral and tibial diameters were significantly different between the male and female participants (*p* < 0.05). The measured widths are listed in Table 3.

Table 6 shows the femorotibial ratios for all the anatomical landmarks. The knee JL (e.g., MEJL) and absolute distances between anatomical landmarks (e.g., IED) were normalized to their femoral or tibial diameters, after which no significant difference was observed between males and females (*p* > 0.05). Since it was impossible to measure the STD/PTFJJL and CTD/TTJL ratios in the same plan as the MRI images, the ratios of these absolute distances were not calculated.

**Table 6:** Femoral and tibial ratios in the study participants (*N* = 130)

Current study				Pereira et al. [9]
Mean ± SD, mm				Mean ± SD, mm
Ratio	Males	Females	Overall	
IED/MEJL	4.3 ± 0.5	4.1 ± 0.5	4.2 ± 0.5	2.8 ± 0.3
IED/LEJL	3.2 ± 0.3	3.2 ± 0.2	3.2 ± 0.2	3.2 ± 0.2
IMD/MFJL	1.9 ± 0.1	2.0 ± 0.2	1.9 ± 0.2	1.7 ± 0.2
IMD/LFJL	2.4 ± 0.2	2.4 ± 0.3	2.4 ± 0.2	2.1 ± 0.2
CTD/PTFJJL	2.5 ± 0.3	2.4 ± 0.3	2.5 ± 0.3	3.3 ± 0.5
STD/TTJL	1.4 ± 0.3	1.4 ± 0.4	1.4 ± 0.3	1.9 ± 0.6

Finally, the absolute femoral and tibial distances were calculated to determine the overall dimensional relationship between the knee JL and femoral and tibial landmarks. As shown in Table 7, LEJL and PTFJJL were equal (LEJL/PTFJJL = 1.0 ± 0.1), suggesting that these landmarks were equally distant from the knee JL. There was no significant difference between males and females in the femoral and tibial ratios (*p* > 0.05).

**Table 7:** Femorotibial ratios in the study participants (*N* = 130)

Mean ± SD, mm			
Ratio	Males	Females	Overall
MEJL/TTJL	0.6 ± 0.1	0.7 ± 0.1	0.7 ± 0.1
LEJL/TTJL	0.9 ± 0.1	0.9 ± 0.1	0.9 ± 0.1
MFJL/TTJL	1.3 ± 0.2	1.3 ± 0.2	1.3 ± 0.2
LFJL/TTJL	1.1 ± 0.2	1.1 ± 0.2	1.1 ± 0.2
MEJL/PTFJJL	0.7 ± 0.1	0.7 ± 0.1	0.7 ± 0.1
LEJL/PTFJJL	1.0 ± 0.1	0.9 ± 0.1	1.0 ± 0.1
MFJL/PTFJJL	1.5 ± 0.2	1.4 ± 0.2	1.5 ± 0.2
LFJL/PTFJJL	1.2 ± 0.1	1.2 ± 0.1	1.2 ± 0.1

## Discussion

This study aimed to determine the location of the knee JL in relation to anatomical landmarks around the knee. Our research using the femorotibial ratio between the LEJL and PTFJ for the identification of anatomical landmarks of the knee JL with a larger patient population generated reliable outcomes. This may help surgeons to perform measurements of the JL in knee replacement for favorable surgical planning, specifically in revision procedures where bone and soft tissue structures are damaged. A surgeon can use a caliber to estimate the joint line intraoperatively by measuring PTFJ and LEJL where the joint line should be equidistant between tham. Moreover, it favored the aim of our study in determining a quantitative reproducible association between the position of the knee JL and recognizable anatomical landmarks around it.

Several studies were carried out to measure knee JL. One study performed at the University of Zurich, Switzerland, used complete preoperative and postoperative radiographs of 22 consecutive patients who underwent TKA. Unfortunately, a mild alteration within the JL occurred from its anatomical location post-primary TKA in these patients. Consequently, the assessment of postoperative primary TKA images is not reliable for JL positioning [14].

Another study conducted in Turkey in 2015 revealed that there was no substantial correlation between the femoral width and the distance from the fibular head to the JL. Moreover, there was a linear correlation, irrespective of factors such as age, sex, and height, between the femoral width and adductor tubercle-JL distance. Therefore, this approach could be considered a dependable landmark for locating the JL level during surgeries [3].

Our analysis showed that the measured ratios for both the femoral and tibial landmarks were constant, which agrees with the findings of a previous study [11]. Our findings related to the measurements of the absolute distances, such as the LEJL (24.4 ± 2.8 mm) and LFJL (30.2 ± 3.5 mm), were similar to those of prior anatomical studies [9, 12, 15].

Several radiographic and anatomical studies were conducted in previous studies to establish a reliable and reproducible method to determine the location of the knee joint, for example, by identifying the correlation between the head of the fibula and tibial plateau; attempts have been limited by the morphological variations and lack of consensus on the reference points of the anatomical landmarks [1, 4, 15]. In this study, the PTFJ approach was found to be superior to the fibular head technique, which has been reported to be unreliable as an anatomical landmark because of the high variability [1, 4, 15]. In contrast to the fibular head, the PTFJ is a clear landmark that can be easily seen on plain radiographs. If it is not visible because of the fibular rotation, it can be found at the intersection of the lateral prominence of the fibular head and fibular styloid [11].

Previous reports have found that LEJL had the lowest SD (2.3 mm), making it the most precise landmark among MFJL, MEJL, PTFJJL, LFJL, and TTJL. Although we found that the MEJL had the lowest SD (2.5 mm), the SD of the LEJL was 2.8 mm in our study, which is still highly comparable to that reported by other studies [13, 16, 17]. Unlike the ME, which is less prominent in the medial distal femur, the LE is the most notable point in the femur. Therefore, the LEJL could serve as a more reliable landmark than the MEJL for accurate measurement of the distances for the knee JL level and the absolute distances equal to 24.4 ± 2.8 mm [11]. Moreover, the MEJL was found to be subject to high intra-observer variability, which is consistent with previous reports [13, 16, 17]. Therefore, it might be an unreliable point to measure on preoperative MR images, as it is a sulcus between two prominences on the medial distal femur, making it difficult to locate it precisely.

Except for TTJL, we found no statistically significant differences between males and females regarding all absolute distances, making them irrelevant in accurately defining knee JL. This difference is supported by the findings of Pereira et al. [11]. Thus, to overcome statistically significant differences between males and females, the authors normalized the absolute distances to their femoral and tibial diameters, which made the calculations more reliable in determining knee JL [11]. Additionally, the use of ratios facilitates the use of the MRI-based method using plain radiographs or computed tomography (CT) scans [11].

Similar to other studies, the investigators found IED/LEJL to be 3.2 ± 0.2 mm (i.e., the LEJL is one-third of the IED), making it a useful and reliable ratio for determining the JL [11]. The measurement of IED/LEJL in this study was also consistent with the results of previous studies that used CT and MRI [9, 12, 15].

The analysis showed that the femorotibial ratio between the LEJL and PTFJ was 1.0 (LEJL/PTFJ = 1.0 ± 0.1), which was first suggested by Pereira et al.; they also suggested that the knee is located at the midpoint between the LE and PTFJ (i.e., two identifiable landmarks) [11].

This study has several limitations. First, the nature of the retrospective study in which bias cannot be eliminated. Second, there might have been a memory effect, as the interval between each measurement was less than 6 weeks. Finally, the measurements of bony landmarks with MRI images may not have been located in the same plane in the most distal and posterior parts, as compared to 3D images. This can present a challenge to surgeons intraoperatively if only bony landmarks on the MRI are used. Therefore, we recommend using anatomical landmarks for preoperative planning in combination with computer-assisted robotics and intraoperative landmarks to achieve a more precise restoration of JL.

## Conclusion

Findings of the current study were comparable to those reported by prior studies, especially the finding that the knee is located at the midline between the LE and PTFJ. This may help surgeons to identify and restore the JL in arthroplasty surgeries. The reproducible quantitative relationships defined in the present work can be broadly employed with different imaging modalities, including CT, MRI, and plain radiography.

## Data Availability

The datasets used and/or analysed during the current study are available from the corresponding author on reasonable request.

## Abbreviations

AP: Anteroposterior
CT: Computed tomography
CTD: Coronal tibial diameter
IED: Interepicondylar diameter
IMD: Intermetaphyseal diameter
JL: Joint line
LE: Lateral epicondyle
LF: Lateral flare
ME: Medial epicondyle
MF: Medial flare
MRI: Magnetic resonance imaging
PTFJ: Proximal tibiofibular joint
ROM: Range of motion
STD: Sagittal tibial diameter
TKA: Total knee arthroplasty
TT: Tibial tubercle
